# Chronic health conditions and mortality among older adults with complex care needs in Aotearoa New Zealand

**DOI:** 10.1186/s12877-023-03961-8

**Published:** 2023-05-22

**Authors:** Rebecca Abey-Nesbit, Hamish A Jamieson, Hans Ulrich Bergler, Ngaire Kerse, John W Pickering, Ruth Teh

**Affiliations:** 1 Department of Medicine, University of Otago, Christchurch, New Zealand; 2 Department of General Practice and Primary Health Care, University of Auckland, M&HS Building 507 - Bldg 507, 28 Park Ave, Grafton, 1850, 1023 Auckland, New Zealand

**Keywords:** Mortality, Ageing, Comorbidities, interRAI, Geriatric assessment

## Abstract

**Background:**

Older people have more comorbidities than younger groups and multimorbidity will increase. Often chronic conditions affect quality of life, functional ability and social participation. Our study aim was to quantify the prevalence of chronic conditions over a three-year period and their association with mortality after accounting for demographics.

**Methods:**

We conducted a retrospective cohort study using routinely collected health data including community-dwelling older adults in New Zealand who had an interRAI Home Care assessment between 1 January 2017 and 31 December 2017. Descriptive statistics and differences between variables of interest among ethnic groups were reported. Cumulative density plots of mortality were developed. Logistic regression models adjusted for age and sex to estimate mortality were created independently for each combination of ethnicity and disease diagnosis.

**Results:**

The study cohort consisted of 31,704 people with a mean (SD) age of 82.3 years (8.0), and of whom 18,997 (59.9%) were female. Participants were followed for a median 1.1 (range 0 to 3) years. By the end of the follow-up period 15,678 (49.5%) people had died. Nearly 62% of Māori and Pacific older adults and 57% of other ethnicities had cognitive impairment. Diabetes the next most prevalent amongst Māori and Pacific peoples, and coronary heart disease amongst Non-Māori/Non-Pacific individuals. Of the 5,184 (16.3%) who had congestive heart failure (CHF), 3,450 (66.6%) died. This was the highest mortality rate of any of the diseases. There was a decrease in mortality rate with age for both sexes and all ethnicities for those with cancer.

**Conclusions:**

Cognitive impairment was the most common condition in community dwelling older adults who had an interRAI assessment. Cardiovascular disease (CVD) has the highest mortality risk for all ethnic groups, and in non-Māori/non-Pacific group of advanced age, risk of mortality with cognitive impairment is as high as CVD risk. We observed an inverse for cancer mortality risk with age. Important differences between ethnic groups are reported.

## Background

In Aotearoa New Zealand it is estimated that the proportion of the total population made up of adults over the age of 65 years will increase from 15% to 2016 to 27% in 2050 [1]. People in older age groups have more comorbidities than younger groups and multimorbidity will increase [2, 3]. Often chronic conditions affect quality of life, functional ability, and social participation [4–6].

While many studies have explored the presence of comorbid conditions amongst adults, they have focused on one specific type of condition rather than a collection of various chronic conditions amongst an older New Zealand cohort [7–9]. The most common causes of death in older age in New Zealand are cancer and cardiovascular disease [10], however it is not clear whether the presence of other conditions is associated with increased mortality.

The New Zealand Health Survey (NZHS) is a key annual survey that identifies the prevalence of conditions among the general population of New Zealand adults however, information about the prevalence of medical conditions in adults aged 65 years and older is unavailable due to the small number of older people in the NZHS [11]. Health data can be useful to understand the population needs and guide cost-effective targeting of treatment and services appropriately [12]. With the ageing population, accurate quantification of prevalence of chronic conditions in advanced age is needed to efficiently target health and social services to those who will benefit most.

New Zealand experiences health disparities among different population groups, like many other countries. These disparities are rooted in social and economic inequalities and can be seen across various health outcomes, including life expectancy, disease prevalence, and access to healthcare. Māori, the indigenous people of Aotearoa New Zealand, experience significant health disparities compared to non-Māori, which persist into advanced age. [13] Pacific peoples disproportionately affected by social and economic inequalities also experience health disparities. For example, they have higher rates of non-communicable diseases (e.g. diabetes and obesity). [11, 14]. Little is known about ethnic-specific information on admission to residential care and health outcomes, which is needed to equip the development of culturally appropriate programmes to mitigate the health and social burden experienced by Māori and Pacific older adults [15].

In a cohort of older people who reached the threshold for complex home services or evaluation for entry into residential care with complex needs, we aimed to quantify the prevalence of chronic conditions and their association with mortality over three years after accounting for demographics and other variables known to be associated with mortality.

## Methods

### Study design

In New Zealand individuals who are referred for consideration of state funded home care support or access to aged residential care undergo a structured assessment using the interRAI Home Care assessment instrument (interRAI-HC). The interRAI-HC has been developed by academics and clinicians from over 30 countries [16, 17]. It is a comprehensive clinical assessment consisting of 236 questions across 20 medical, functional and social domains [16, 17]. Assessments are conducted by trained clinical assessors and data aggregated in a national database held in a central repository. We conducted a retrospective cohort study using routinely collected health data from the interRAI-HC (version 9.1) assessment [18]. De-identified interRAI-HC assessment data was obtained from Technical Advisory Services in Aotearoa New Zealand (TAS). Mortality information was obtained from the Ministry of Health’s Mortality Collection register [19] and matched to the interRAI-HC data using an encrypted unique identifier.

### Participants

Study participants were older adults in Aotearoa New Zealand living at home, with complex care needs—the sample consisted of everyone aged 65 years and older, 55 years and older for Māori and Pacific Peoples [18], who had an interRAI-HC assessment from 1 January 2017 to 31 December 2017. Where an individual had more than one assessment in 2017, their first assessment for the year was used. Participants were followed until the end of the study period (30 June 2020) or death if that occurred prior to the end of the study period. The study was approved by the Health and Disability Ethics Committee (14/STH/140/AM07), the interRAI is mandated in NZ, and only those who consented for their data to be used for research purposes were included in the study cohort; 95.7% of people undergoing the interRAI-HC in 2017 gave consent (Technical Advisory Services, 2020).

### Variables

A previously published New Zealand study exploring patterns of multi-morbidity and mortality among community-dwelling octogenarians [20], and availability of data in the interRAI-HC informed the selection of disease diagnoses to be included in this study. Details about assessment process and coding instructions can be found in the user manual for the interRAI-HC assessment [18]. The disease diagnoses included were Alzheimer’s disease, dementia other than Alzheimer’s disease, coronary heart disease, congestive heart failure, chronic obstructive pulmonary disease (COPD), diabetes mellitus, cancer, stroke/cerebrovascular accident (CVA), and depression. All disease diagnoses in the interRAI-HC assessment are determined with the use of available clinical records [21]. Responses to disease diagnoses within the interRAI are: “not present”, “primary diagnosis/diagnosis for current stay”, “diagnosis present, receiving active treatment”, and “diagnosis present, monitored but no active treatment”. For analysis purposes we condensed response options into “no diagnosis” and “diagnosis present”.

We defined cognitive impairment as the presence of Alzheimer’s disease, or of dementia other than Alzheimer’s disease, or a cognitive performance scale (CPS) [21] of 2 or higher. The CPS measures an individual’s level of cognitive impairment based on items such as memory, decision-making skills, and level of consciousness [21], scores range between 0 and 6. Additionally, we defined depression as having a diagnosis of depression or had a depression rating scale [22] score of 3 or higher. The Depression Rating Scale (DRS) is a tool to identify people who may have depression, scores range between 0 and 14, and the measure is based on questions such as repetitive anxious behaviours, making negative statements, and persistent anger with self or others [22]. We chose to combine the DRS and depression diagnosis because depressive states can vary over time and we wanted to include those who had depressive symptoms at the time of their assessment.

### Statistical analysis

**Fig. 1:**
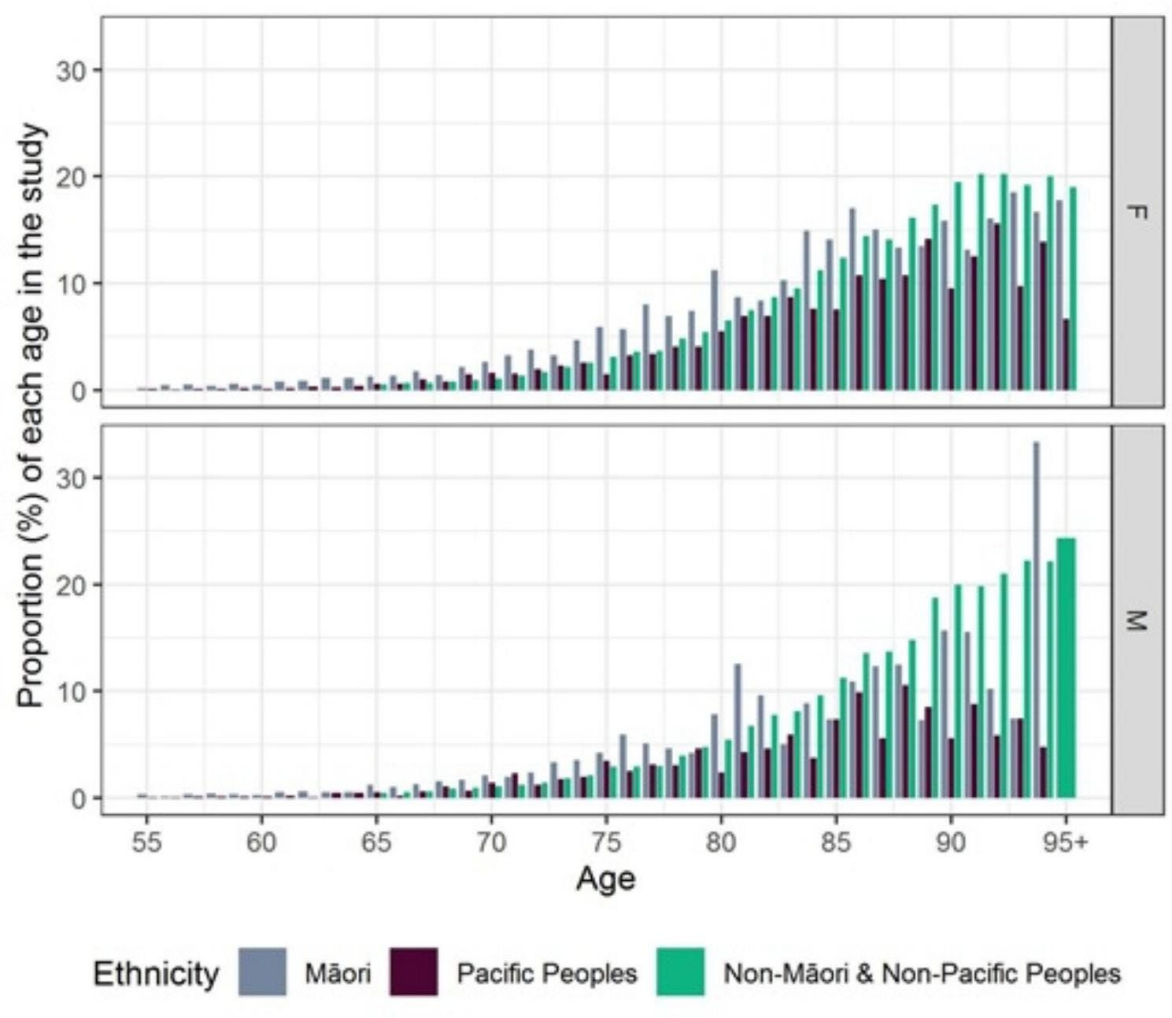
Proportion of the NZ population for each age that was represented in the study by sex and ethnicity For each ethnicity and age, the proportion of the New Zealand population of each ethnicity and age who are included in the study F = female, M = male

Descriptive statistics were reported for variables of interest (age, ethnicity, sex, marital status, cognitive impairment, coronary heart disease, congestive heart failure, COPD, diabetes mellitus, cancer, stroke/CVA, and depression), including stratified by survival status. Additionally, differences between variables of interest among Māori and Pacific Peoples and the non-Māori and Pacific Peoples were reported in view of the health disparities observed between these ethnic groups [11, 14]. We adopted the New Zealand Ministry of Health ethnicity prioritisation protocol and presented the data separately for Māori, Pacific Peoples, and other ethnicities. Prioritised ethnicities are such that if an individual lists Māori as one of several ethnicities then their priority ethnicity is Māori irrespective of any other ethnicity. [23] We compared our cohort to the 2018 New Zealand Estimated Resident Population (NZ-ERP) [24] across different age groups and sex for each ethnicity and show this as proportions (Fig. 1). Cumulative density plots were developed for mortality. Logistic regression models adjusted for age and sex to predict mortality were created independently for each combination of ethnicity and disease diagnosis. We chose only these two covariates as they are two of the six variables most closely associated with mortality in this cohort [25]. Three of the other variables are co-morbidities in this study (Cancer and CHF) or highly correlated with them (dyspnoea). The other variable, BMI, is missing for many patients, so it was decided to not include it. One-hundred replicates for each model were created using 100 bootstrapped, with replacement, data sets and included in the figure to illustrate the precision of the estimated association between age and chronic condition for each ethnicity and sex.

Statistical analysis was conducted using IBM SPSS Statistics version 27 (IBM Corp, Armonk, NY), and all the models for figures were created with R version 4.0 (R Foundation for Statistical Computing, Vienna, Austria) using the base stats and the ggplot packages.

Reporting of analyses adhered to the Strengthening the Reporting of Observational Studies in Epidemiology (STROBE) guidelines [26]. Ethics approval was granted from the Ministry of Health’s Health and Disability Ethics Committee (14/STH/140/AM07).

## Results

The study cohort consisted of 31,704 people 7.1% identified as Māori, 3.8% Pacific peoples and 89.1% NZ European or other ethnicity. More than half of participants were female (59.9%), (Table 1). The cohorts mean (SD) age was 82.3 (8.0) years and differed across the different ethnic groups with 75.1 (SD 9.1) years for Māori, 77.3 (SD 8.4) for Pacific Peoples and 83.1 (SD 7.5) for Non-Māori/non-Pacific people. The proportions are contrasted by mortality and the greatest mortality was in the 85 + age groups.

**Table 1:** Demographic and disease diagnosis incidence totals, and stratified by survival status with follow up until 30 June 2020 or death

	Total N (%)	Alive N (%)	Died N (%)
	31,704	16,026 (50.5)	15,678 (49.5)
**Age (Years)**			
Mean (SD)	82.3 (8.0)		
55–64	415 (1.3)	251 (1.6)	164 (1.0)
65–74	5,088 (16.0)	3,183 (19.9)	1,905 (12.2)
75–84	12,271 (38.7)	6,808 (42.5)	5,463 (34.8)
85–94	12,616 (39.8)	5,419 (33.8)	7,197 (45.9)
95+	1,314 (4.1)	365 (2.3)	949 (6.1)
**Ethnicity**			
Māori	2,263 (7.1)	1,240 (7.4)	1,023 (6.3)
Pacific	1,219 (3.8)	737 (4.4)	482 (2.9)
Non-Māori non-Pacific	28,222 (89.1)	14,703 (88.1)	14,834 (90.8)
**Sex** ^**a**^			
Female	18,997 (59.9)	10,614 (66.2)	8,383 (53.5)
Male	12,684 (40.0)	5,403 (33.7)	7,281 (46.4)
**Marital Status** ^**b**^			
Never married	1,535 (4.8)	849 (5.3)	686 (4.4)
Married/civil union/defacto	12,649 (39.9)	6,198 (38.7)	6,451 (41.1)
Widowed	14,467 (45.6)	7,231 (45.1)	7,236 (46.2)
Separated	803 (2.5)	457 (2.9)	346 (2.2)
Divorced	1,843 (5.8)	1,067 (6.7)	776 (4.9)
Other	306 (1.0)	173 (1.1)	133 (0.8)
**Cognitive Impairment**	18,109 (57.1)	8,730 (54.5)	9,379 (59.8)
**Coronary heart disease**	10,259 (32.4)	4,625 (28.9)	5,634 (35.9)
**Congestive heart failure**	5,184 (16.4)	1,734 (10.8)	3,450 (22.0)
**Chronic obstructive pulmonary disease**	5,135 (16.2)	2,137 (13.3)	2,998 (19.1)
**Diabetes mellitus**	6,739 (21.3)	3,282 (20.5)	3,457 (22.1)
**Cancer**	5,142 (16.2)	1,630 (10.2)	3,512 (22.4)
**Stroke/Cerebrovascular accident**	5,506 (17.4)	2,715 (16.9)	2,791 (17.8)
**Depression**	7,835 (24.7)	4,128 (25.8)	3,707 (23.6)
**Number of conditions, median (IQR)**	2 (1;3)	2 (1;3)	2 (1;3)
0 conditions	2,791 (8.8)	1,881 (11.7)	910 (5.8)
1 conditions	9,025 (28.5)	5,159 (32.2)	3,866 (24.7)
2 conditions	9,822 (31.0)	4,908 (30.6)	4,914 (31.3)
3 conditions	6,258 (19.7)	2,682 (16.7)	3,576 (22.8)
4 + conditions	3,808 (12.0)	1,396 (8.7)	2,412 (15.4)

^a^23 values missing, ^b^101 values missing

For Non-Māori and Non-Pacific the highest proportions of the NZ age population of men in the sample were in their 90s and that proportion increased through the 90s. For Non-Māori and Non-Pacific females the proportion of the NZ age population were highest in the 90s where the proportion plateaued at ~ 20% (Fig. 1). Pacific peoples had a lower representation at all age groups than the other ethnicities, and this reached a maximum in the mid-80s. The proportion of the total NZ Māori age population that appeared in the HC sample was higher at younger ages than other ethnicities, but lower at older ages. Overall, as age increased, greater proportions of all ethnic groups appeared in the interRAI sample.

The cohort was followed for a median 1.1 (range 0 to 3) years. By the end of the follow-up period 49.5% people had died. More than half of people across all three ethnic groups had cognitive impairment (Table 2). Diabetes mellitus was the next most prevalent amongst Māori (38.4%) and Pacific Peoples (47.8%), and coronary heart disease was most prevalent amongst Non-Māori/non-Pacific individuals (32.5%).

**Table 2:** Demographic and disease diagnosis incidence totals, stratified by ethnic group

	Māori N(%)	Pacific Peoples N(%)	Non-Māori/Non-Pacific N (%)
**Total**	2,263	1,219	28,222
**Age (Years)**			
Mean (SD)	75.1 (9.1)	77.3 (8.4)	83.1 (7.5)
55–64	322 (14.2)	93 (7.6)	N/A
65–74	681 (30.1)	347 (28.5)	4,060 (14.4)
75–84	903 (39.9)	518 (42.5)	10,850 (38.4)
85–94	341 (15.1)	255 (20.9)	12,020 (42.6)
95+	16 (0.7)	6 (0.5)	1,292 (4.6)
**Sex**			
Female	1,425 (63.0)	725 (59.5)	16,847 (59.7)
Male	838 (37.0)	494 (40.5)	11,375 (40.3)
**Marital Status**			
Never married	180 (8.0)	71 (5.9)	1,284 (4.6)
Married/civil union/defacto	676 (30.0)	487 (40.2)	11,486 (40.8)
Widowed	979 (43.4)	550 (45.5)	12,938 (46.0)
Separated	170 (7.5)	41 (3.4)	592 (2.1)
Divorced	160 (7.1)	47 (3.9)	1,636 (5.8)
Other	89 (3.9)	14 (1.2)	203 (0.7)
**Cognitive Impairment**	1,399 (61.8)	742 (60.9)	15,968 (56.6)
**Coronary heart disease**	802 (35.4)	274 (22.5)	9,183 (32.5)
**Congestive heart failure**	556 (24.6)	190 (15.6)	4,438 (15.7)
**Chronic obstructive pulmonary disease**	630 (27.8)	194 (15.9)	4,311 (15.3)
**Diabetes mellitus**	869 (38.4)	583 (47.8)	5,287 (18.7)
**Cancer**	313 (13.8)	123 (10.1)	4,706 (16.7)
**Stroke/Cerebrovascular accident**	387 (17.1)	291 (23.9)	4,828 (17.1)
**Depression**	489 (21.6)	190 (15.6)	7,156 (25.4)
**Number of conditions, median (IQR)**	2 (1;3)	2 (1;3)	2 (1;3)
0 conditions	104 (4.6)	71 (5.8%)	2,616 (9.3)
1 conditions	504 (22.3)	318 (26.1)	8,203 (29.1)
2 conditions	659 (29.1)	418 (34.3)	8,745 (31.0)
3 conditions	553 (24.4)	262 (21.5)	5,443 (19.3)
4 + conditions	443 (19.6)	150 (12.3)	3,215 (11.4)

**Fig. 2:**
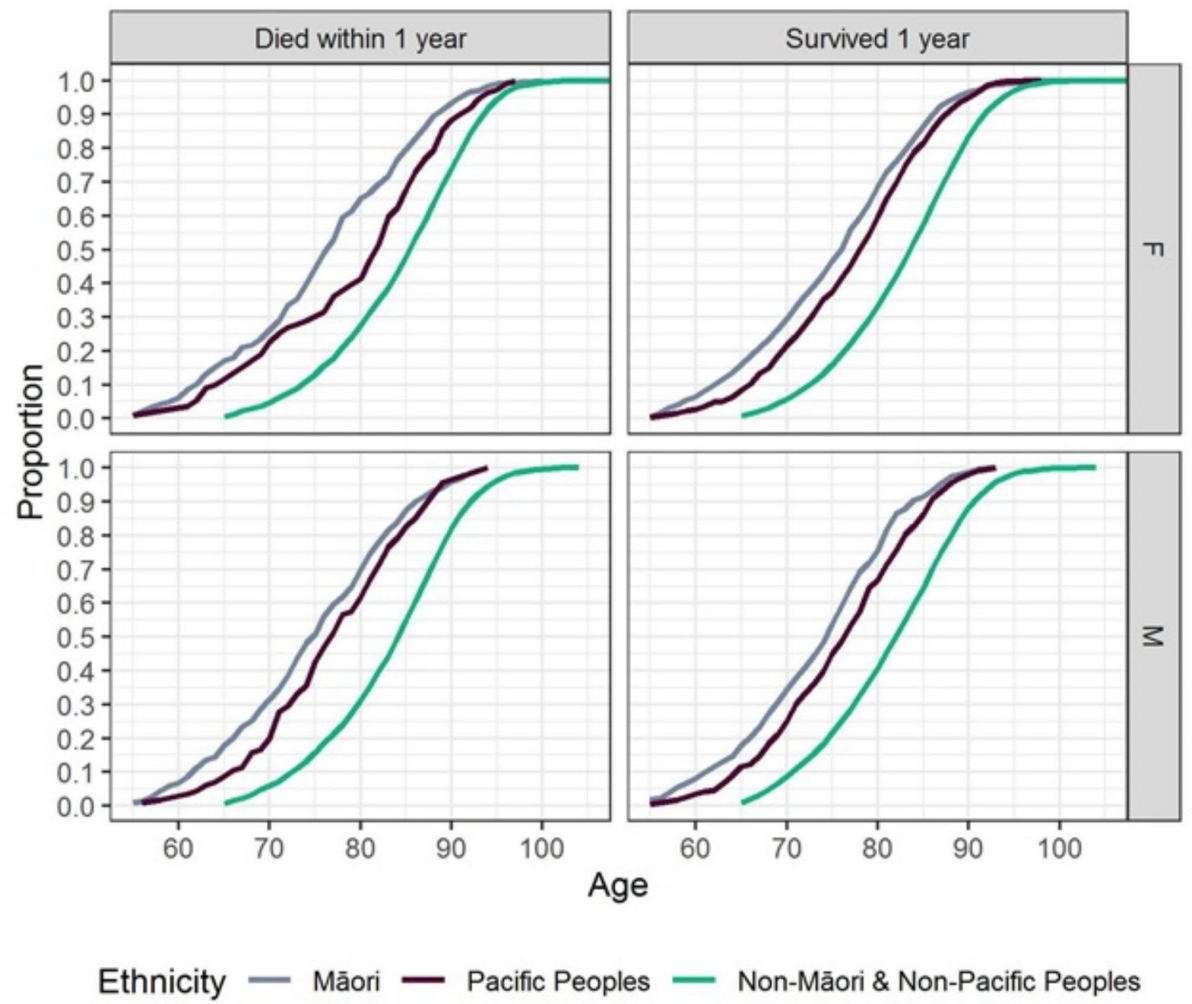
Cumulative density plots of mortality by age among different ethnic groups Stratified by sex, ethnicity, and survival status to one year the cumulative proportions of participants in the study. This may be read two ways. e.g. (1) In the bottom left panel for Males who died, a vertical line from 80 intersects the Non-Māori/non-Pacific curve at 0.3 indicating 30% of Non-Māori/non-Pacific under the age of 80 died within 1 year. (2) The age by which 30% of Māori Males have died is approximately 69. F = female, M-Male.

Within 1-year 30% of Non-Māori Non-Pacific individuals under the age of 80 had died comparatively the age by which 30% of Māori males died was 69, and 71 for Pacific males, (Fig. 2). For female non-Māori non-Pacific individuals 30% of people under the age of 80 had also died within 1 year, however, the age by which 30% of Māori females died was approximately 71 and Pacific females was approximately 75 as shown in (Fig. 2).

**Fig. 3:**
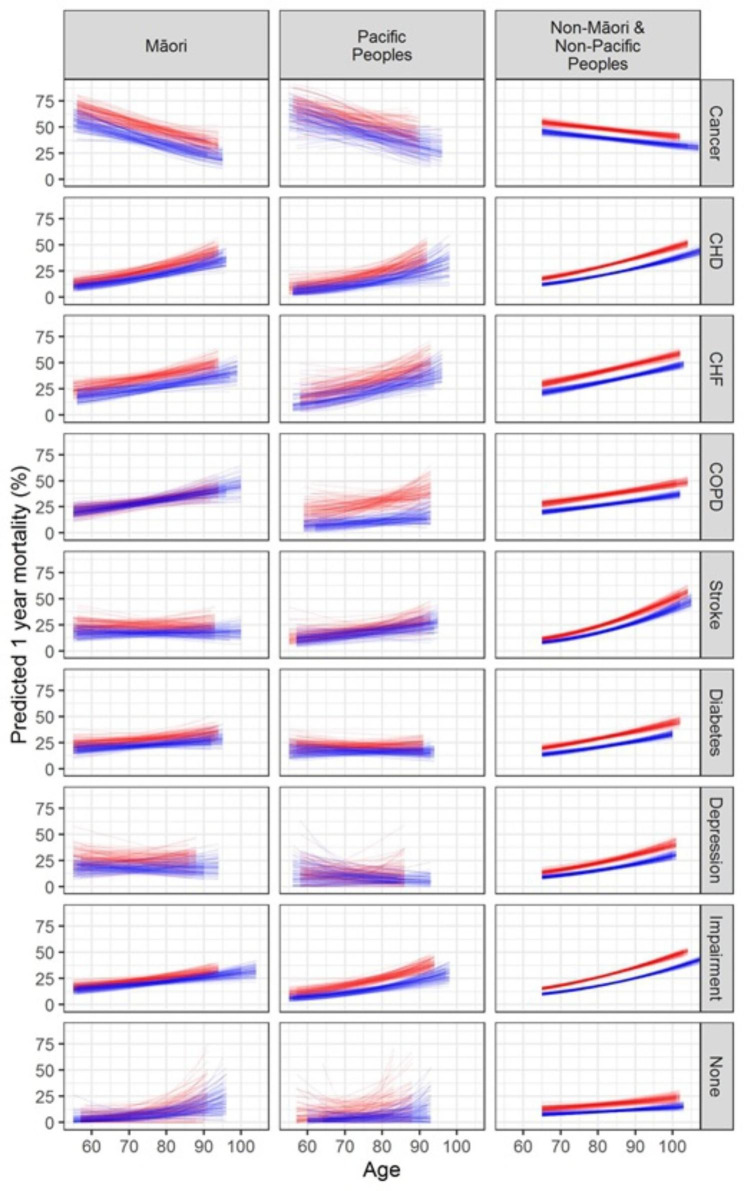
Relationship between age, chronic conditions and mortality stratified by ethnicity and sex Red are females, blue are males. Each line represents the fit to one of 100 bootstrapped samples of predicted mortality for each ethnic group with each condition. Each graph represents the 100 fits within the sub-group defined by ethnicity and disease status adjusted for age and sex. Impairment = Cognitive impairment; CHD = Coronary Heart Disease; CHF = Congestive Heart Failure; COPD = Chronic Obstructive Pulmonary Disease; None = none of the preceding conditions

The highest mortality rate was observed among the 5,142 (16.2%) with a diagnosis of cancer, where 3,512 (68.3%) had died by the end of the study period Table 1. Cancer has highest mortality risk but the risk decreases with age (Fig. 3). This observed trend is in reverse compared to other conditions, i.e. as age increased the risk of predicted 1-year mortality increased for each ethnic group and each condition (Fig. 3). Māori with COPD and Pacific Peoples with depression, stroke or diabetes had similar risk of mortality between males and females. For Non-Māori Non-Pacific individuals, stroke had the highest risk of 1-year mortality, particularly for those in older age. For older aged Pacific Peoples, those who had COPD had the highest risk of 1-year mortality, and in Māori those who had CHD had the highest risk of 1-year mortality.

## Discussion

Our study quantified the prevalence of chronic conditions and their association with mortality in Māori, Pacific and non-Māori non-Pacific older people referred and assessed for publicly funded support services. The presence of conditions and outcomes related to conditions was different in each of the ethnic groups.

The prevalence of congestive heart failure and coronary heart disease was higher in Māori than in Pacific and Non-Māori/Non-Pacific people, consistent with previous literature documenting disparities in the prevalence of cardiovascular disease among Māori, Pacific and European communities [13, 27, 28]. Inequalities have been shown to be declining [29], however our results suggest they currently persist in this population group of those requiring support services in older age. We add that there are higher prevalence of diabetes for Māori and Pacific people and stroke for Pacific people in this group, than in other New Zealand studies identifying Pacific Peoples health conditions, [14, 30] probably because this study examines rates in those with established disability requiring support services. Cognitive impairment was the most prevalent condition in Māori, Pacific and Non-Māori/Non-Pacific people. The proportion of Non-Māori/Non-Pacific people with cognitive impairment in the (57%) was lower than a Canadian home-care sample (80%) of similar age (mean age 82.8 years) [31]. However, with an increasing proportion of the older population in all ethnic groups in the next five decades [32], the risk of conversion from cognitive impairment to dementia [33] highlights the importance of appropriate approaches to mitigate the risk of cognitive impairment and associated risk factors [34].

This study showed there are differences in the proportion of Māori and Pacific Peoples receiving interRAI assessments at different ages (Fig. 1). The results suggest that access and use of the interRAI assessment process differs across age groups with appropriate higher access for younger groups of Māori and Pacific Peoples potentially due to a higher morbidity burden. We observed fewer assessments in older Māori compared to the general population level, aligning with previous findings that either health services are not accessed or earlier mortality among Māori and Pacific Peoples. This may be attributed to inequity in support service use, barriers to accessing support use, or the type of support available is less acceptable [35].

There were also differences between the mortality proportion among Māori, Pacific, and non-Māori non-Pacific people. Figure 2 reflects that older Māori and Pacific peoples are more likely to die at any given age than older Non-Māori/non-Pacific Peoples. This may be reflections of higher morbidity burden and differential access to health and support services across the lifespan.

Older people have the right to accessible and appropriate support services. This study emphasises that this does not necessarily happen equally in New Zealand. We reiterate the importance of looking at ethnic groups separately to identify whether access to assessment is equitable and see mortality risk factors that may otherwise be hidden by the majority group, non-Māori and non-Pacific individuals in the cohort. The interRAI-HC database is large enough that we are able to make valid comparisons between different ethnic groups. Pathways to interRAI assessment may vary across Aotearoa New Zealand along with expectations of care and support. Earlier access to standardised interRAI assessments may help address barriers to access to appropriate health services. The New Zealand government has increased focus on inequity in health so as to better align health resources to improve health outcomes for those currently most disadvantaged.

Our study has many strengths including a large standardised dataset that covers a large array of clinical, social, and other domains. The data collected is on a national level, and assessments are conducted using trained assessors. However, there are also some limitations. The cohort consisted of older adults with complex health care needs who have been identified as requiring publicly funded support or long-term aged care services, therefore these findings may not be generalisable to the general population of older adults in New Zealand. Mortality rates in this group with establish disability requiring support services will be higher than a population-based sample and should be interpreted as similar to other groups of older people receiving home care services or being considered for aged residential care placement. The disease diagnoses information was limited to what is available in the assessment and may not reflect detailed clinical information. For example, while cancer is listed as one of the diagnoses of interest, information about the type of cancer the individual has is not included in the interRAI assessment data. While Asian and Middle East Latin America Africa (MELAA) are increasing in numbers the respective numbers are small, and these groups are culturally very diverse. A pragmatic approach for this study aim is that we have grouped Asian ethnicities with non-Māori/non-Pacific people as they have similar life-expectancy. We recommend future InterRAI research include more in-depth analysis of Asian and MELAA ethnicities to provide nuances on each group access interRAI assessments. We are not able to comment on conditions or mortality of those not coming forward for assessment and reasons for not being assessed will be a complex mix of culturally bound patterns of care, choice, and access barriers within the health and social care system. This will be the focus of ongoing research hoping to improve equity in outcomes for all New Zealanders.

## Conclusions

In community dwelling older adults who had an interRAI assessment, cognitive impairment was the most common condition, but cancer has the highest mortality risk albeit decreases with age. In this paper we took a single disease approach to describe the population, and examine mortality risk of specific conditions, further research could explore how the presence of multiple chronic conditions can have differing effects on outcomes.

## Data Availability

The data that support the findings of this study are available from Te Whatu Ora - Health New Zealand by emailing interRAI@tas.health.nz but restrictions apply to the availability of these data, which were used under license for the current study, and are not publicly available.

## List of abbreviations

CHF: Congestive Heart Failure
COPD: Chronic Obstructive Pulmonary Disease
CPS: Cognitive Performance Scale
CVA: Cerebrovascular accident
DRS: Depression Rating Scale
interRAI-HC: International residential assessment instrument home care
NZ-ERP: New Zealand Estimated Resident Population
NZHS: New Zealand Health Survey
SD: Standard Deviation
STROBE: Strengthening the Reporting of Observational Studies in Epidemiology
TAS: Technical Advisory Services
